# The complex relationship between antibody titers and clinical outcome in botulinum toxin type A long-term treated patients with cervical dystonia

**DOI:** 10.1007/s00415-022-11235-3

**Published:** 2022-07-17

**Authors:** Harald Hefter, Beyza Ürer, Raphaela Brauns, Dietmar Rosenthal, Sven G. Meuth, John-Ih Lee, Philipp Albrecht, Sara Samadzadeh

**Affiliations:** Department of Neurology, University of Düsseldorf, Moorenstrasse 5, 40225 Düsseldorf, Germany

**Keywords:** Cervical dystonia, Course of the disease, Botulinum toxin therapy, Long-term outcome, Secondary treatment failure, Antibody formation

## Abstract

**Background:**

Repeated injections with abo- or onabotulinumtoxin type A (aboBoNT/A, onaBoNT/A) may lead to induction of neutralizing antibodies (NABs) and/or a secondary treatment failure (STF). The relation between NABs and STF is still unclear.

**Aim of the study:**

To demonstrate that a significant improvement can be observed in patients with STF after abo- or onaBoNT/A-treatment when switched to incobotulinumtoxin type A (incoBoNT/A) and that in NAB-positive patients without STF abo- or onaBoNT/A-treatment can be continued without significant worsening.

**Methods:**

Paralysis times (PT) of the mouse hemidiaphragm assay (MHDA) and clinical outcome (TSUI-score) was analyzed in 60 patients with cervical dystonia (CD) and STF after abo- or onaBoNT/A-treatment (STF-group) who were switched to incobotulinumtoxin type A (incoBoNT/A). These data were compared to those of 34 patients who were exclusively treated with incoBoNT/A (INCO-group). Furthermore, PTs and TSUI-scores were followed up over 7 years in 9 patients with NABs but without STF who were switched to inco-BoNT/A (SWI-group) and 9 other patients with NABs who remained on their previous BoNT/A preparation (NO-SWI-group).

**Results:**

In the STF-group, a significant improvement of TSUI-scores could be detected after switch to incoBoNT/A. This improvement was less pronounced than in the INCO-group. There was no significant difference in long-term outcome between the SWI- and NO-SWI-group.

**Conclusion:**

The best strategy is to avoid the induction of NABs. A switch to incoBoNT/A may lead to improvement in patients with STF. However, in some patients with NABs without STF, BoNT/A-treatment can be continued without significant worsening.

## Introduction

Injections with botulinum neurotoxin type A (BoNT/A) are an effective and safe treatment for a variety of neurological and non-neurological indications [1, 2]. For most indications, BoNT/A has to be applied repeatedly to maintain a stable plateau of improvement [1]. However, repeated injections of the 150 kDa large BoNT/A molecule implicate the risk of neutralizing antibody (NAB) formation.

It seems to be common sense that NAB formation occurs only in a small percentage of BoNT/A-treated patients [3, 4]. Unfortunately, in many studies reporting antibody rates or prevalence of NABs, antibody tests are only performed in selected patients. However, by definition, it is necessary to test all patients in a cohort, to determine the prevalence of a symptom, especially of the presence of NABs. Thus, the prevalence of NABs in a cohort can only precisely be determined by a cross-sectional study [5].

So far, only a few cross-sectional studies on NAB formation in BoNT/A long-term treated patients are available. In still-responding patients with cervical dystonia (CD) [6], and in another study on CD-patients and 5 other indications [7], high prevalences of NABs of more than 11% have been reported. Both studies emphasize the dependence of NAB formation on dose per session and duration of treatment [6, 7].

Prevalence divided by mean duration of treatment allows a rough estimation of the incidence of NAB formation. The incidences reported so far for ona- and abobotulinumtoxin type A (onaBoNT/A, aboBoNT/A) vary between 0.5 and 2.5%/year [6–11]. Taking into account that, in clinical practice, BoNT/A-treatment is performed up to 40 years, high rates of NAB-positive patients have to be expected in long-term BoNT/A-treatment.

Nevertheless, the relationship between secondary non-responsiveness or secondary treatment failure (STF) and NABs is unclear [3, 4]. The difficulty on the one hand is that STF is not precisely defined. STF due to an insufficient dose, inappropriate muscle selection, or improper injection technique is not a secondary treatment failure in the strict sense, but a suboptimal treatment. When such sub-optimally treated patients are tested for the presence of NABs, a large percentage turns out to be negative in the mouse hemidiaphragma assay (MHDA) [12]. This is most likely the reason why 53.5% of patients with a “STF” do not have positive MHDA-tests [12, 13]. In still-responding patients, the restriction of NAB-testing to selected patients is the most likely reason why “prevalence” has been reported to be as low as 3.5% [13].

On the other hand, in clinical practice, in many centers, the outcome is not or barely routinely controlled by means of simple assessment scales (− 1 = worsened, 0 = no change, and 1 = improved) which are insensitive to subtle changes of outcome. This is similar to the result of the mouse lethal assay (MLA) which is either positive or negative. Such discrete parameters are not suitable for correlation analysis between clinical findings and NAB-testing.

But even when well-established scales as the TSUI-score [14] or the CDQ24-questionnaire [15] are used for therapy monitoring, the correlation between these clinical outcome measures and the paralysis time, the outcome measure of the MHDA, is low [5, 16, 17].

To contribute further to the analysis of this complex relationship between clinical outcome and antibody titres, the present study was performed correlating the results of MHDA-testing and treatment-related data of a cross-sectional study on 60 clinically well-characterized CD-patients with STF who had been switched to incobotulinumtoxinA (incoBoNT/A). Furthermore, a small, longitudinal study is added comparing MHDA-test results and clinical data in 18 still-responding CD-patients who had been tested in 2010, 2013, and 2017. Nine of these 18 patients had been switched to incoBoNT/A, 9 remained on their previous BoNT/A preparation even after the detection of NABs.

## Methods

The present study was performed according to the guidelines for good clinical practice (GCP) and according to the declaration of Helsinki. It was approved by the local ethics committee of the University of Düsseldorf (number: 4085). The presented data are parts of the medical theses of Beyza Ürer (BÜ) and Raphaela Brauns (RB).

### Patients (STF-group, SWI-group, NO-SWI-group)

In 2017, BÜ screened more than 120 charts of patients with CD, who were regularly treated in the outpatient department of the University hospital at Düsseldorf (Germany) and in whom their initial BoNT/A preparation had been switched to another BoNT/A preparation during their course of treatment in Düsseldorf because of the development of a partial or complete secondary treatment failure (switchers). Switchers who fulfilled the following inclusion and exclusion criteria were informed on the purpose of the study while they were waiting for the next BoNT/A in the botulinum toxin ambulance.

Inclusion criteria of the study were: (i) age over 17, (ii) diagnosis of idiopathic CD, (iii) continuous treatment in the botulinum toxin ambulance every 12–13 weeks without interruption of BoNT therapy of more than one treatment cycle, and (iv) at least 3 injections of BoNT. Exclusion criteria were: (i) patient under legal care, (ii) multifocal, segmental, and/or symptomatic dystonia at the onset of BoNT therapy, and (iii) additional disabling disease other than CD. Especially, patients with clinical manifest disturbances of mood and perception were excluded.

Sixty patients gave informed written consent and were consecutively recruited (STF-group). Depending on the initial BoNT/A preparation (aboBoNT/A or onaBoNT/A), switchers were split up into the ABO-group (*n* = 48) and the ONA-group (*n* = 11). In one patient, the initial BoNT/A preparation had not been documented.

In 2017, RB screened more than 100 charts of CD-patients, who had been treated with incoBoNT/A. Those patients who fulfilled the above-mentioned inclusion and exclusion criteria were informed on the purpose of the study. 34 of these patients gave written informed consent and were consecutively recruited (INCO-group).

During the screening of the charts, BÜ detected nine still-responding CD-patients who had participated in a cross-sectional study in 2010 [6] and had decided to continue their BoNT/A therapy as before, although they had had a positive MHDA-test (non-switchers). These nine patients (NO-SWI-group) were compared to nine other patients who also had been MHDA-tested in 2010, and had been switched to incoBoNT/A either immediately before or after the antibody test in 2010 (SWI-group). These 18 patients had also been MHDA-tested in 2013. These 18 patients were included into a small longitudinal study. Data collection was identical to data collection in the cross-sectional study.

### Treatment-related data and outcome measures

Patients in our BoNT ambulance are trained to assess the remaining severity of CD in percent of the severity of CD at onset of BoNT therapy every day. For each month, either a prepared table on a sheet of paper or an Exel^®^-table with 31 rows (corresponding to the days of the month) and 21 columns ranging from 0 up to 100% in 5% steps had to be completed.

On the day of recruitment, patients were asked for the change of the severity of CD in percent of the severity of CD at the onset of BoNT/A therapy (IMPQ). Furthermore, they had to mark the actual severity of CD (ASCD) on a line of about 15 cm length with 0 indicating complete relief of symptoms and 10 cm indicating the severity of CD at the onset of BoNT/A therapy. A value shorter than 10 indicated improvement, and a value larger than 10 indicated worsening. The value 10*(10-ASCD) was the percentage of the change of the severity of CD calculated from this visual analogue scale (IMPD). The treating physician scored the actual severity of CD at the day of recruitment by means of the TSUI-score (14; ATSUI) and documented the BoNT/A preparation used as well as the actual total dose (ADOSE).

The following demographical data were extracted from the charts: age at the day of recruitment (AGE), age at onset of symptoms (AOS), age at onset of therapy (AOT), and duration of therapy (DURT). The time span during which patients had tolerated symptoms without BoNT therapy was determined (DURS = AOT-AOS). BÜ and RB also extracted the best TSUI-score (BTSUI) documented in the charts of the recruited patients and the time span from the onset of symptoms to BTSUI (TTB).

TSUI-score at onset of therapy (ITSUI), initial BoNT-preparation, and initial total dose (IDOSE) were extracted from the charts. Improvement of the severity of CD on the basis of the TSUI-score (IMPTSUI) was calculated as (ITSUI-ATSUI) *100/ITSUI). For sake of comparison, doses of different preparations were transformed into unified dose units (uDU) by multiplying ona- and incoBoNT/A doses by 3 and leaving aboBoNT/A doses unchanged following evidence-based data and a European consensus paper [18]. The increase of dose (INDOSE) during treatment was calculated as ADOSE-IDOSE.

Alternative therapies as acupuncture, physiotherapy, etc. were not controlled in the present study.

### Antibody testing

On the day of recruitment, blood samples were taken and deeply frozen. After recruitment of all patients, all blood samples (*n* = 112 = 60 + 34 + 18) were coded and sent to a blinded contractor (Toxogen^®^ Lab., Hannover, Germany) for MHDA-testing. All samples were analyzed in a batch and a complete list of MHDA-test results (paralysis times) was returned.

### Statistics

Patients were split up into an ABO-, ONA-, and INCO-subgroup. A Chi-square analysis was performed to whether sex distribution and NAB frequency were different across patient subgroups. A three-group repeated measurement (rm)-ANOVA was performed to test whether AGE, AOS, DURS, DURT, IDOSE, ADOSE, INDOSE, ITSUI, BTSUI, ATSUI, IMPTSUI, IMPQ, and IMPD were different between the subgroups. Pearson and Spearman’s rho correlation coefficients were determined for the paralysis time and clinical data (TSUI-scores, IMPD, IMQ). Register operational characteristics (ROC)-curves of IMPQ, IMPD, IMPTSUI, ATSUI, and ADOSE analyzing sensitivity and specificity for MHDA-positivity were calculated. All statistical analyses were performed using the SPSS^®^ statistics package (version 25; IBM, Armonk, USA).

## Results

### Patient’s and physician’s assessment of the treatment effect

**Fig. 1:**
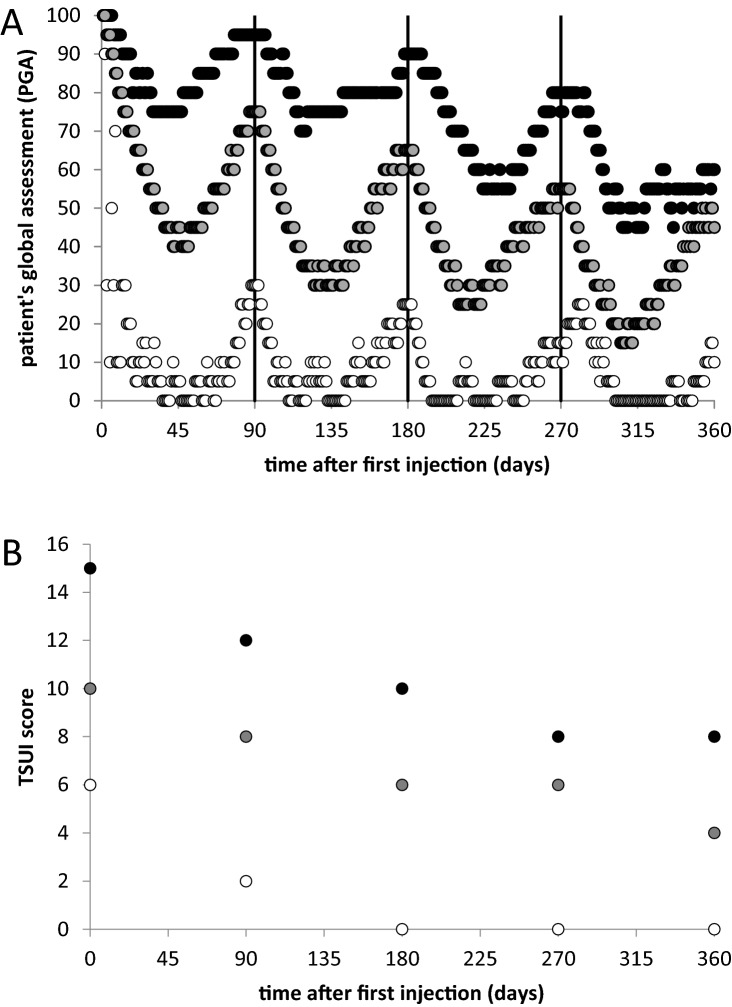
**A** Patient’s assessment of the efficacy of the first 4 incobotulinumtoxin injections of a CD-patient with secondary treatment failure after previous abobotulinumtoxin treatment (full circles), a moderately responding de-novo CD-patient (grey dots), and a golden responder (de novo-CD-patient) with the excellent response already to the first injection (open circles). With repeated injections, every 3 months a stable plateau is approached which may differ from patient to patient. **B** TSUI-scores of the same three patients as in **A** determined before each of the first 5 incoBoNT/A injections of these 3 patients

Patients were trained to assess the effect of treatment by scoring daily the severity of CD in percent of the initial severity of CD at the onset of BoNT therapy (patient’s global assessment (PGA)). Three examples of PGA-curves of the first 4 incoBoNT/A injections are presented. The black dots in Fig. 1A correspond to PGA-values of a 53-year-old male patient who had developed a complete secondary treatment failure after initially successful aboBoNT/A-treatment over years at another center. He was switched to incoBoNT/A and documented an improvement in percent of the severity of CD at the onset of incoBoNT/A. The second patient is a moderately affected typical 45-year-old female de novo-CD-patient (Fig. 1A; grey dots) and the third a mildly affected 61-year-old female de novo-CD-patient (Fig. 1A; open dots) of the INCO-group who scored the effect of incoBoNT/A injections in percent of the initial severity at onset of BoNT therapy. The vertical bars indicated days 90, 180, and 270 around which incoBoNT/A injections had been performed. In Fig. 1B, TSUI-scores determined by the same physician of all three patients are presented. The “golden responder” (patient 3: open circles) reached a stable plateau of 0 already after the first two injections. In the other two patients (grey and black dots), the severity of CD decreased stair-case like even after the first four injections.

### Best outcome (BTSUI) in the three subgroups who were exclusively treated with one of the three licensed BoNT/A preparations

**Fig. 2:**
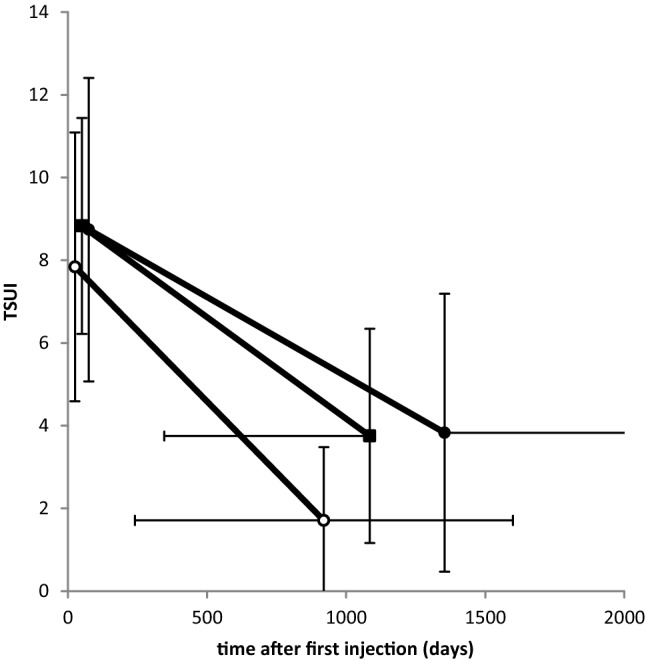
Comparison of the mean initial TSUI-score (ITSUI) and the mean best TSUI-score (BTSUI) in CD-patients who were exclusively treated with incoBoNT/A (open circles), with onaBoNT/A (full squares), and with aboBoNT/A (full circles). The vertical bars indicate standard deviations of ITSUI resp. BTSUI and the horizontal bars indicate standard deviations of the time to best TSUI (TTB). Only the difference between BTSUI of the aboBoNT/A and incoBoNT/A-treated patients was significant (*p* < 0.05)

The initial severity of CD (ITSUI) at the onset of BoNT therapy was about the same in the ABO- and ONA-group (ABO-group (full circles in Fig. 2): mean 8.74, S.D. 3.67; ONA-group (full squares in Fig. 2): mean 8.82, S.D. 2.61) and non-significantly lower in the INCO-group (open circles: mean 7.84, S.D. 3.25). Initial doses (ONA-group: mean: 210 U Botox^®^ (SD 113); ABO-group: mean 620 U Dysport^®^ (SD 150); INCO-group: mean 189 U Xeomin^®^ (SD 73) did not differ significantly. With ongoing BoNT therapy, the TSUI-score significantly (*p* < 0.001) improved in all three patient subgroups.

Mean best TSUI-score (BTSUI) was the lowest (1.7/S.D. 1.7) in the INCO-group (open circles in Fig. 2) and was reached after 30.7 months in the mean (S.D. 22.6 months). BTSUI was the highest (3.8/S.D. 3.4) in the ABO-group (full circles in Fig. 2), and was reached after 45 months in the mean (S.D. 45 months). The difference of mean BTSUIs of these two groups was significantly different (*p* < 0.005). In the small ONA-group (full squares), mean BTSUI was 3.7 and reached after 36 months (S.D. 24.6 months). Doses had been increased, but did not differ at TTB across the three patient groups (ONA-group 321 U Botox^®^ (S.D. 229); ABO-group: 843 U Dysport*®* (S.D. 697); INCO-group: 267 U Xeomin^®^ (S.D. 90). In all three subgroups, TTB was highly variable and the shortest in the INCO- and the longest in the ABO-group. Because of the high variability, TTB did not differ significantly between the three patient subgroups.

### Development of a secondary treatment failure in the ABO- and ONA-group and improvement after switch to incoBoNT/A

After an initial good response, the switchers in the ABO- and ONA-group developed a partial or complete secondary treatment failure and severity of CD worsened again until patients were switched to incoBoNT/A. After 7.9 years of aboBoNT/A-treatment in the mean (S.D. 5.6 years), mean TSUI increased up to 7.8 (S.D. 3.1). After 7.3 years of onaBoNT/A-treatment in the mean (S.D. 4.3 years), mean TSUI increased up to 8.4 (S.D. 3.6). On the day of switch to incoBoNT/A, TSUI-score (STSUI) did not significantly differ from ITSUI in the ABO- and in the ONA-group. Initial incoBoNT/A dose in the ONA- (mean: 245 U Xeomin^®^ (S.D. 82)) and that in the ABO-group (mean: 265 U Xeomin^®^ (S.D. 73) were nearly identical to BDOSE in the INCO-group.

After 7.2 years (S.D. 3.1 years) of incoBoNT/A-treatment, incoBoNT/A dose had been increased to 349 U Xeomin^®^ (S.D. 92) in the ABO-group. The TSUI-score decreased significantly (*p* < 0.005) to 5.32 (ATSUI; S.D. 2.56). In the ONA-group, incoBoNT/A dose had been increased to 370 U Xeomin^®^ (S.D. 87) after 8.8 years (S.D. 2.7 years). The TSUI-score significantly (*p* < 0.05) decreased to 6.82 (ATSUI; S.D.: 4.2).

After 6.3 years of incoBoNT/A therapy (S.D. 2.3), incoBoNT/A dose was increased to 305 U Xeomin^®^ (S.D. 91). ADOSE did not differ between the three patient groups. Mean ATSUI was 3.3 (S.D. 2.4) in the INCO-group which is even lower than mean BTSUI and by far lower than mean ATSUI in the ABO- and ONA-group.

### Analysis of paralysis times in the incoBoNT/A- and in the abo- and naBoNT/A group

On the day of recruitment, only 1 patient (9.1%) was MHDA-positive in the ONA-group and 14 patients (= 26.1%) were MHDA-positive in the ABO-group. In the INCO-group, no patient had a positive MHDA-test. Chi^2^-testing yielded a significant difference between the prevalence of MHDA-positive patients in the ABO- compared to the INCO-group, but not between ONA- and INCO-group or ABO- and ONA-group.

**Fig. 3:**
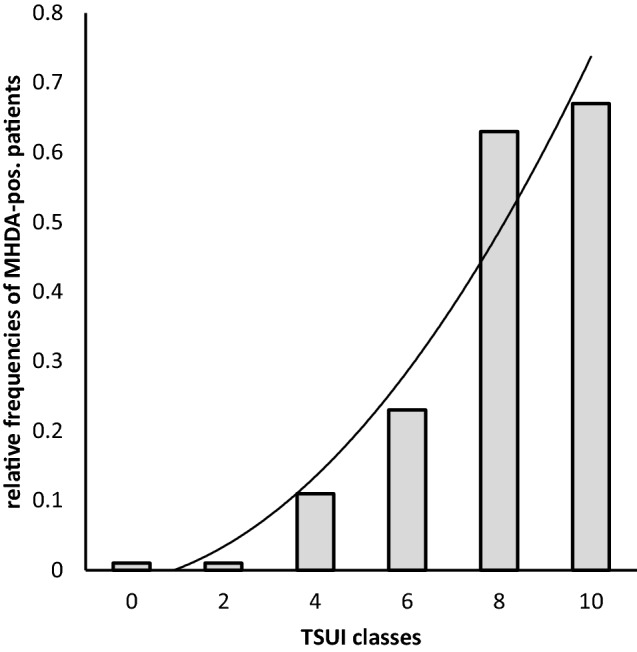
Relative frequencies of MHDA-positive patients in TSUI-ranges of 2 TSUI-score point widths

**Fig. 4:**
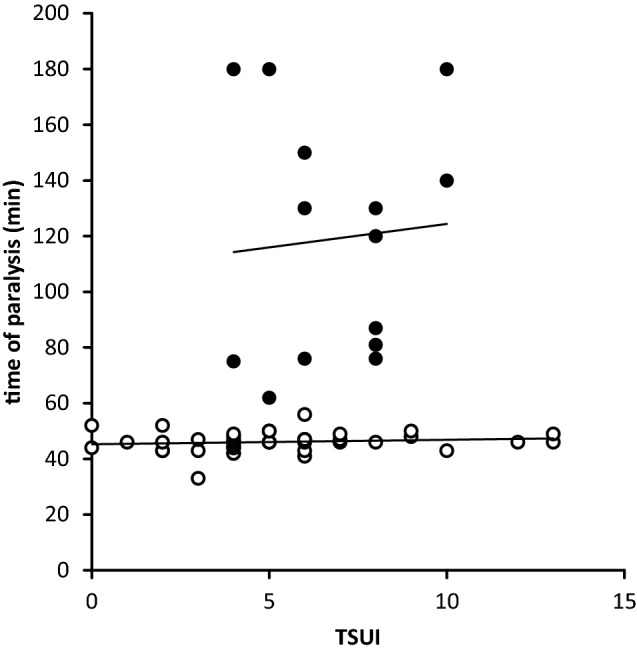
Correlation between the time to paralysis (ordinate) and TSUI-score (abscissa) in the MHDA-negative patients (open circles) and the MHDA-positive patients (full circles)

When relative frequencies of MHDA-positive patients in the entire STF-group (ABO- plus ONA-group) were calculated for TSUI-ranges with a bin-width of 2 TSUI-points a non-linear increase of the prevalence of MHDA-positive patients was found up to a TSUI-range of 11–12 (Fig. 3). Above this range and in the low TSUI-ranges (< 4), no patient was MHDA-positive (see Figs. 3 and 4).

When PT and TSUI-scores were correlated in the MHDA-negative switchers (open circles in Fig. 4), a flat regression line was found (0.16*TSUI + 45.28; *r* = 0.135, *p* = 0.393, n.s.). In the MHDA-positive switchers (full circles in Fig. 4), the regression line was different, but also without a significant increase (1.685*TSUI + 107.52; *r* = 0.078, *p* = 0.791, n.s.). When the regression line was calculated for all patients, a clear tendency, but not a significant correlation was found (3.00*TSUI + 47.42; *r* = 0.227, *p* = 0.092, n.s.). However, Spearman’s rho rank correlation coefficient between PT and TSUI-score was significant (*r* = 0.362, *p* < 0.006) for the entire STF-group. Good or even better non-parametric correlations were found between PT and IMPQ or IMPD (IMPD: *r* = − 0.359, *p* < 0.006; IMPQ: *r* = 0.383, *p* < 0.003).

### Analysis of sensitivity and specificity (ROC-curves) of various parameters indicating the presence of NABs

**Fig. 5:**
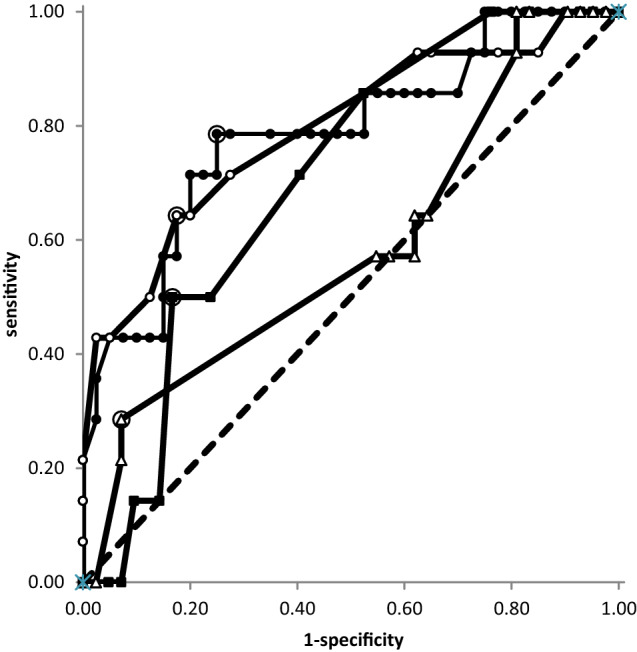
ROC-curves for the analysis of MHDA-positivity: IMPQ (full circles), IMPD (open circles), ATSUI (full squares), and unified dose per session (open triangles). The patient’s report of improvement (IMPQ) predicts positivity in the MHDA-test best

In Fig. 5, ROC-curves are presented analyzing the sensitivity/specificity relation for MHDA-positivity of four parameters (IMPQ, IMPD, ATSUI, and ADOSE). The best sensitivity (0.792) for MHDA-positivity was observed for patient’s report of percentage of improvement of CD (IMPQ) (full circles in Fig. 5). The second-best sensitivity was observed for the improvement drawn by the patient on a VAS (open circles; IMPD 0.787). The actual severity of CD (ATSUI, full squares in Fig. 5) also showed a sensitivity to the presence of antibodies (0.706). Less sensitive was the improvement of the TSUI-score (not shown in Fig. 5; IMPTSUI 0.614) and the dose per session (open triangles in Fig. 5; ADOSE 0.587). The presence of NABs is likely, when IMPQ is smaller than 39.6%, when IMPD is smaller than 32.5%, when ATSUI is larger than 7.5, when IMPTSUI is smaller than 24%, and when ADOSE is larger than 1230 uDU.

### Development of TSUI, dose, and PT in the patients of the follow-up study

**Fig. 6:**
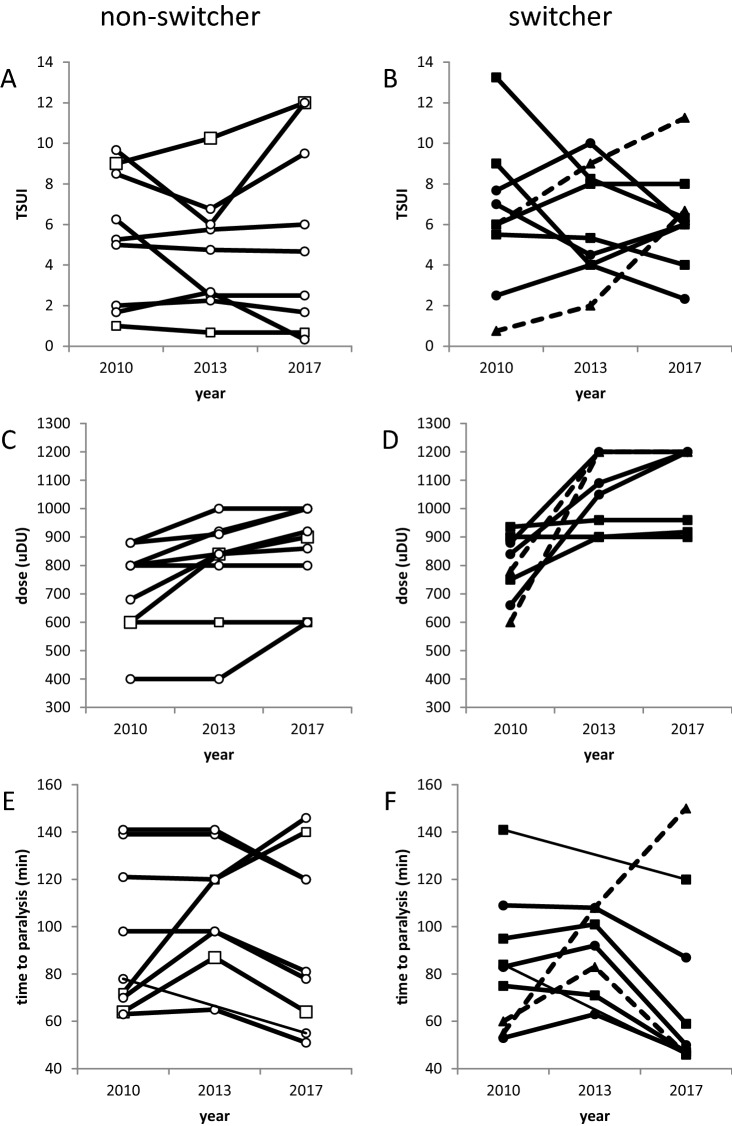
Temporal course of the TSUI-score (A,B; upper part), dose per session (C,D: middle part), and paralysis time (E,F; lower part) between 2010 and 2017 in the non-switchers (left part) and in the switchers (right part). Oopen squares in A,C,E indicate 2 onaBoNT/A-treated patients and open circles 7 aboBoNT/A-treated patients. Full squares in B,D,F indicate patients being switched from ona- to incoBoNT/A and full circles indicate patients being switched from aboBoNT/A to incoBoNT/A. Full lines in B,D,F indicate patients being switched in 2010 before MHDA-test, and hatched lines indicate patients being switched in 2010 after the MHDA-test

In the year 2010, a cross-sectional study on still-responding CD-patients had been performed [6]. Nine patients who had had a positive MHDA-test in 2010 after abo- (*n* = 7; circles in Fig. 6A) or onaBoNT/A therapy (*n* = 2; squares in Fig. 6A) had decided to continue BoNT/A therapy as before. They were retested in 2013 and 2017. In these 9 non-switchers (NO-SWI-group), mean TSUI remained fairly constant between 2010 and 2017 (2010: mean 5.37, S.D. 3.10; 2013: mean 4.62, S.D. 2.77; 2017: mean 5.48, S.D. 4.41), the dose per session was mildly increased (2010: mean 716, S.D. 150; 2013: mean 794, S.D. 174; 2017: mean 853, S.D. 150), and the paralysis time of the MHDA-test (PT) remained fairly constant in the mean (2010: mean 94, S.D. 30.1; 2013: mean 108.5, S.D. 24.6; 2017: mean: 95.0, S.D. 34.7) (Fig. 6, left side).

The results of these nine non-switchers could be compared to the results of 9 patients in the SWI-group who had received a MHDA-test in 2010 and had been switched to incoBoNT/A in 2010 either before (*n* = 7) or after (*n* = 2; hatched lines in Fig. 6 right side) the MHDA-test. They had also been retested in 2013 and 2017. In these nine switchers (SWI-group), mean TSUI remained constant between 2010 and 2017 (2010: mean 6.41, S.D. 3.40; 2013: mean 6.12, S.D. 2.60; 2017: mean 6.29, S.D. 2.33), the dose per session was significantly increased (2010: mean 805, S.D. 110; 2013: mean 1044, S.D. 127; 2017: mean 1075, S.D. 140), and the paralysis time of the MHDA-test (PT) slightly decreased (2010: mean 83.9, S.D. 26.8; 2013: mean 89.4, S.D. 16.5; 2017: mean 72.4, S.D. 36.3) (Fig. 6 right side).

In a two group rm-ANOVA, no significant differences between the NO-SWI- and the SWI-group could be detected.

### Correlations between TSUI and PT in the follow-up study

**Fig. 7:**
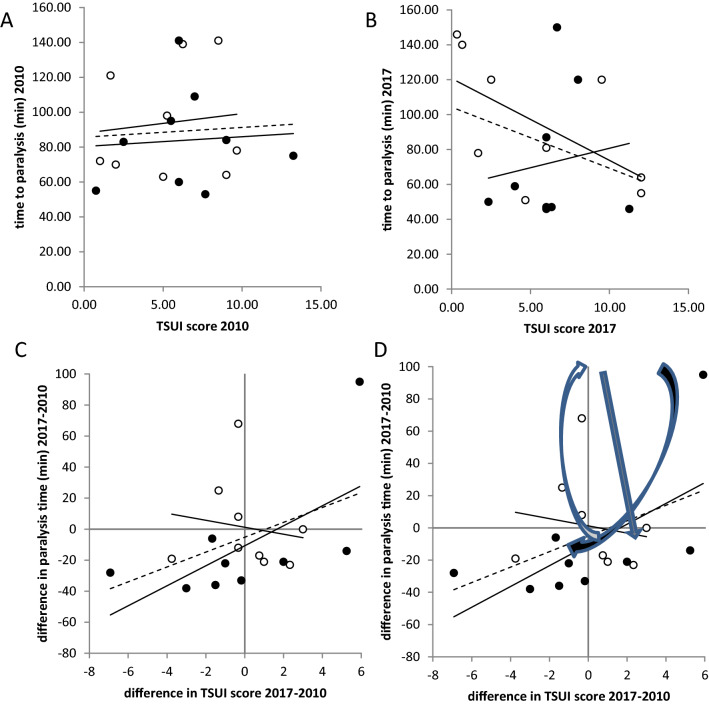
Correlation between the change of the paralysis time (ordinate) and TSUI-score (abscissa) between 2010 and 2017 in the non-switcher (open circles) and the switcher (full circles). The trend line of the non-switchers (full line) and trend curve of the switchers (dotted second-order polynomial). **A** Correlation between paralysis time (ordinate) and TSUI-score (abscissa) in the 18 patients of the follow-up study in 2010 (open circles = non-switcher; full circles = switcher). The hatched line indicates the corresponding regression line between paralysis time and TSUI-scores of all 18 patients. **B** Correlation between paralysis time (ordinate) and TSUI-score (abscissa) in the 18 patients of the follow-up study in 2017 (open circles = non-switcher; full circles = switcher). The hatched line indicates the corresponding regression line between paralysis time and TSUI-scores of all 18 patients. **C** Correlation between the differences of paralysis times 2017–2010 (ordinate) and differences of TSUI-scores 2017–2010 (abscissa) in the 18 patients of the follow-up study (open circles = non-switcher; full circles = switcher). The hatched line indicates a significant (*p* < 0.05; 1-sided testing) increase. **D** Same data as in **C**. The light arrow indicates an initial increase of NAB titers and TSUI-scores. The grey arrow indicates a possible decrease of NAB titers without change or even increase of TSUI-score. The dark arrow indicates decrease of NAB titers followed by a decrease of TSUI-scores when patients with NABs were switched to incoBoNT/A. Thus, the arrows indicate the complex relationship between NAB titers and clinical outcome

Correlation between TSUI-scores (x-axis in Fig. 7A) and paralysis times of the year 2010 (PT; y-axis in Fig. 7) in the 9 non-switchers (open circles: 1.12*TSUI + 87.97; *r* = 0.116, n.s.) and in the 9 switchers (full circles: 0.56*TSUI + 80.27; *r* = 0.071, n.s.) yielded regression lines which looked very similar and had a positive slope. The regression line between TSUI-score and PT in all 18 patients (hatched line in Fig. 7A) did not show a significant increase (0.56*TSUI + 85.67; *r* = 0.063, n.s.). This corresponds to the missing correlation between TSUI-scores and PT in the entire STF-group (see results part 4).

Correlation between TSUI-scores and PT of the year 2017 (Fig. 7B) in the 9 non-switchers (− 4.70*TSUI + 120.75; *r* = − 0.597, *p* < 0.05 (1-sided testing)) yielded a negative slope, whereas in the 9 switchers (2.23*TSUI + 58.40; *r* = 0.143, n.s.) a positive slope. The regression line between TSUI-score and PT in all 18 patients (hatched line in Fig. 7B) showed a non-significant decrease (− 3.51*TSUI + 104.35; *r* = − 0.334, n.s.).

Correlation of the changes of PT (PT2017-PT2010) with TSUI-scores of the year 2010 yielded a significantly negative correlation (non-switchers: *r* = − 0.833; *p* < 0.01; switchers: *r* = − 0.260; n.s.; all: *r* = − 0.647, *p* < 0.0037).

Correlation of the differences of the TSUI-score between 2017 and 2010 and the differences of the paralysis times (PT2017-PT2010; Fig. 7D) for all 18 patients (hatched line in Fig. 7) yielded a positive correlation was found (4.79*(TSUI2017-TSUI2010)-5.20; *r* = 0.417 (p < 0.05, 1-sided testing) which was even more pronounced in the switchers (6.44*(TSUI2017-TSUI2010) + 10.66; *r* = 0.628; *p* < 0.05, 1-sided testing), whereas the corresponding regression line in the non-switchers had a negative slope (− 2.24(TSUI2017-TSUI2010) + 1.25; *r* = − 0.151, n.s.).

As a consequence, we think that changes of antibody titers follow a hysteresis: first, the antibody titers increase, then the TSUI-score worsens (open arrow in Fig. 7D). When the BoNT-preparation is switched, first, the NAB titers decrease and then the TSUI-scores (full arrow in Fig. 7D). The grey arrow in Fig. 7D indicates that a decrease of NAB titers may occur but without clear improvement of severity of CD when the BoNT/A preparation is not switched.

These data indicate that a complex relation exists between NAB titers and clinical outcomes during ongoing BoNT treatment.

## Discussion

### Variability and availability of data on clinical long-term outcome

There is a long-standing debate whether secondary treatment failure is caused by NAB induction or not [for a recent review, see [3]]. In full agreement with other authors, we think that an unsatisfactory treatment effect reported by the patient may have a variety of reasons [19, 20]. Patient’s report on a reduced efficacy of BoNT/A-treatment is a sensitive indicator for the presence of NABs as demonstrated in Fig. 5. However, this does not exclude other reasons for the reduced efficacy of BoNT injections as progression of the disease severity [20] and that the treatment effect can be improved by adaption of injection scheme and dose and the use of ultrasound or electromyography guidance [19, 20, 23].

We therefore have based the present study on a cross-sectional antibody testing in clinically well-characterized patients with a clear improvement measured by means of the TSUI-score in the beginning of the BoNT therapy and a second clear worsening later on also measured by means of the TSUI-score.

The analysis of the relationship between clinical outcome and NAB titers relies both on the quality of the clinical data and the quality of the laboratory NAB-testing. In clinical practice, disease severity or improvement after the onset of BoNT therapy is usually not quantified or scored in detail in most cases. Often, only improvement, no change, or worsening is documented corresponding to a 3-point Lickert scale [22]. This lack of solid information on clinical long-term outcomes is a major reason why little information on the correlation between antibody titers and clinical outcomes has been presented so far. In MHDA-positive, still-responding CD-patients who had been tested in a cross-sectional study [6, 24] and had been scored by means of the TSUI-score [6] and the CDQ24 [24] yielded a significant correlation between the paralysis time and the unified dose units and between the paralysis time and the pain subscore of the CDQ24 were found [5]. In the present paper analyzing the presence of NABs in CD-patients in whom the BoNT/A preparation had been switched during the course of treatment, a significant non-parametric correlation (p < 0.006) between the TSUI-score and the paralysis time was detected. The ROC-curve analysis demonstrated that patient’s report on the improvement of CD since the onset of incoBoNT/A therapy indicated the presence of NABs even more significantly (p < 0.003) and better than the ATSUI score scored by the treating physician (Fig. 5).

This underlines that there is a relationship between clinical outcome and antibody titers, but because of the lack of or the variability of the clinical data, it is difficult to detect this relationship.

### Availability of follow-up data of NABs

The analysis of the relationship between clinical outcome and NAB titers also relies on the quality of the laboratory NAB-testing. The re-test reliability of the MHDA-test is high as long as a single blood sample of the same patient is analyzed [25]. However, little is known about the test/re-test reliability of serial blood samples over months. To our knowledge, there are only a few follow-up studies on NAB-titres available. A decline of NAB titers is described after cessation of BoNT therapy [26]. A similarly steep decrease of NAB titers has also been observed after switching from treatment with a complex protein containing to a complex protein-free BoNT/A preparation [16].

Apart from the above-mentioned data of Dressler and Bigalke [26], data on a long-term follow-up of NAB titers in still-responding patients without a switch of BoNT/A preparation are not available. It is surprising to see that patients may still respond to a BoNT-preparation over years (Fig. 6, left side), although NABs are present. A mild increase of dose per session may be sufficient to maintain the level of response over years (Fig. 6, left side).

The mouse lethality assay (MLA) does not provide appropriate information on antibody titers and does not allow the calculation of a parametric correlation between clinical outcome and antibody titers. Furthermore, the technical details of the MHDA are difficult to maintain constant over years. Therefore, it is difficult to compare antibody titers over years.

These are further reasons why it is difficult to demonstrate a significant relationship between clinical outcomes and NAB titers.

### Weak correlation between the severity of CD and NAB titers

It is difficult to detect a significant correlation between the paralysis time as an outcome measure of the MHDA and clinical outcome measures. However, the more refined the clinical outcome measure is, the more significant is this relationship. Patient’s assessment of the improvement was documented on a scale with 21 steps between 0 and 100%, and physicians rated the severity of CD by means of the TSUI-score which usually varies between 4 and 16 with a worse resolution to detect subtle changes of disease severity. This explains why the sensitivity to predict a positive MHDA-test result of IMPQ or IMPD was higher than that of ATSUI (Fig. 5) and IMPTSUI.

### Effect of switching the BoNT/A preparation on clinical outcome and NABs

The MHDA-test reliably detects a decrease of NAB titers when NABs have been induced under abo- or onaBoNT/A and BoNT/A therapy is switched to incoBoNT/A. This has been demonstrated in a previous study [16, 17], is demonstrated in Fig. 7, and has also been observed by others [23]. The clinical improvement in parallel to the decrease of NAB titers is less convincing (Fig. 7C).

This is poorly understood. Whether cellular immunological reactions to BoNT therapy may play a role in addition to antibody-mediated immunological processes has not been excluded so far. And whether a botulinum neurotoxin molecule can still be taken up into a presynaptic nerve terminal of a mouse muscle despite a human antibody being bound to a special epitope of the BoNT molecule has also not been excluded. This would imply that human NAB titers were determined or estimated too low in the MHDA.

As demonstrated in Fig. 7D, CD severity and NAB titers seem to be regulated according to a complex hysteresis relationship. First, NABs increase (Fig. 7C; open arrow in Fig. 7D), and then, severity worsens, so that no NAB-positive patients are found in low (< 4) TSUI-classes (Fig. 3 and 4). Then, the NAB titers decline again without much change of severity (grey arrow in Fig. 7D). When the preparation is switched, first NAB titers decline (black arrow in Fig. 7D) before the clinical severity improves. These findings underline the complexity of the relationship between clinical outcome and NAB titer.

### Strengths and limitations of the present study

The present study describes the largest cohort of patients with secondary treatment failure in whom BoNT/A preparation is switched from a complex containing to a complex free BoNT/A preparation. Furthermore, it presents data on the follow-up of NAB titers in still-responding patients in whom BoNT/A preparation has not been switched. A highly significant correlation between NAB titers and clinical outcomes was detected. NABs and clinical outcome possibly follow a complex hysteresis regulation process. However, this is highly speculative. We, therefore, recommend a well-designed multi-center long-term study in future to analyze the temporal development of clinical outcome and NAB induction and to provide a better data basis for the understanding of the difficulty to demonstrate a significant relationship between NAB titers and clinical outcome.

## Data Availability

The data that support the findings of this study are available from the corresponding author, Prof. Harald Hefter, upon reasonable request.

## Abbreviations

aboBoNT/A: Abobotulinum neurotoxin type A
ABO-group: Patients with STF after aboBoNT/A-treatment
ADOSE: BoNT/A dose at recruitment
AGE: Age at recruitment
AOS: Age at onset of symptoms
ATSUI: TSUI-score at recruitment
BoNT: Botulinum neurotoxin
BoNT/A: Botulinum neurotoxin type A
BTSUI: Best TSUI during BoNT treatment
CD: Cervical dystonia
DURS: Duration of symptoms before onset of BoNT therapy
DURT: Duration of therapy
IDOSE: Initial BoNT/A dose
IMPD: Improvement according to visual analogue scale
IMPQ: Improvement according to a questionnaire
IMPTSUI: Improvement according to changes of the TSUI-score
incoBoNT/A: Incobotulinum neurotoxin type A
INCO-group: Patients being exclusively treated with incoBoNT/A
INDOSE: Increase of BoNT/A dose
ITSUI: Initial TSUI-score
MHDA: Mouse hemidiaphragma assay
NABs: Neutralizing antibodies
NO-SWI-group: Group of 9 patients without switch of BoNT/A preparation who received NAB-testing 2010,2013 and 2017
onaBoNT/A: Onabotulinum neurotoxin type A
ONA-group: Patients with STF after onaBoNT/A-treatment
PGA: Patients global assessment
PT: Paralysis time
STF: Secondary treatment failure
STF-group: Entire cohort of the study
SWI-group: 9 Patients in whom BoNT/A was switched and who received NAB-testing in 2010, 2013, and 2017
TSUI: TSUI-score

## Code availability

SPSS version 24 and ACCESS data bank of the research center of university hospital Duesseldorf.

## References

[1] Simpson DM, Hallett M, Ashman EJ (2016). Practice guideline update summary: botulinum neurotoxin for the treatment of blepharospasm, cervical dystonia, adult spasticity, and headache: report of the Guideline Development Subcommittee of the American Academy of Neurology. Neurology.

[2] Royal College of Physicians (2018). BSoRM. Spasticity in adults: management using botulinum toxin. National guidelines.

[3] Bellows S, Jankovic J (2019). Immunogenicity associated with botulinum toxin treatment. Toxins.

[4] Bellows S, Jankovic J (2019). Reply to comment on re-visiting immunogenicity associated with botulinum toxin reatment. Toxins.

[5] Hefter H, Rosenthal D, Bigalke H, Moll M (2019). Clinical relevance of neutralizing antibodies in botulinum toxin long-term treated still-responding patients with cervical dystonia. Ther Adv Neurol Disord.

[6] Hefter H, Rosenthal D, Moll M (2016). High botulinum toxin-neutralizing antibody prevalence under long-term cervical dystonia treatment. Mov Disord Clin Pract.

[7] Albrecht P, Jansen A, Lee JI (2019). High prevalence of neutralizing antibodies after long-term botulinum neurotoxin therapy. Neurology.

[8] Kessler KR, Skutta M, Benecke R (1999). Long-term treatment of cervical dystonia with botulinum toxin A: efficacy, safety, and antibody frequency. J Neurol.

[9] Mejia NI, Vuong KD, Jankovic J (2005). Long-term botulinum toxin efficacy, safety, and immunogenicity. Mov Disord.

[10] Brin MF, Comella CL, Jankovic J, Mmath FL, Naumann M (2008). Long-term treatment with botulinum toxin type A in cervical dystonia has low immunogenicity by mouse protection assay. Mov Disord Off J Mov Disord Soc.

[11] Kranz G, Sycha T, Voller B, Kranz GS, Schnider P, Auff E (2008). Neutralizing antibodies in dystonic patients who still respond well to botulinum toxin type A. Neurology.

[12] Lange O, Bigalke H, Dengler R, Wegner F, deGroot M, Wohlfarth K (2009). Neutralizing antibodies and secondary therapy failure after treatment with botulinum toxin type A. Clin Neuropharmacol.

[13] Fabbri M, Leodori G, Fernandes RM (2016). Neutralizing antibody and botulinum toxin therapy: a systematic review and meta-analysis. Neurotox Res.

[14] Tsui JC, Jon Stoessl A, Eisen A, Calne S, Calne D (1986). Double-blind study of botulinum toxin in spasmodic torticollis. Lancet.

[15] Müller J, Wissel J, Kemmler G (2004). Craniocervical dystonia questionnaire (CDQ-24): development and validation of a disease-specific quality of life instrument journal of Neurology. Neurosurg Psychiatry.

[16] Hefter H, Hartmann C, Kahlen U, Moll M, Bigalke H (2012). Prospective analysis of neutralizing antibody titres in secondary non-responders under continuous treatment with a botulinumtoxin type A preparation free of complexing proteins-a single cohort 4-year follow-up study. BMJ Open.

[17] Hefter H, Hartmann CJ, Kahlen U, Samadzadeh S, Rosenthal D, Moll M (2021). Clinical improvement after treatment with incobotulinumtoxinA (XEOMIN^®^) in patients with cervical dystonia resistant to botulinum toxin preparations containing complexing proteins. Front Neurol.

[18] Contarino MF, van den Dool J, Balash Y (2017). Clinical practice: evidence-based recommendations for the treatment of cervical dystonia with botulinum toxin. Front Neurol.

[19] Ferreira JJ, Colosimo C, Bhidayasin R (2015). Factors influencing secondary non-response to botulinum toxin type A injections in cervical dystonia. Park Rel Disord.

[20] Jinnah HA, Goodmann E, Rosen AR (2016). Botulinum toxin treatment failures in cervical dystonia: causes, management, and outcomes. J Neurol.

[21] Erro R, Bhatia KP, Esposito M, Cordivari C (2016). The role of polymyography in the treatment of cervical dystonia. J Neurol.

[22] Moll M, Rosenthal D, Hefter H (2018). Quality of life in long-term botulinum toxin treatment of cervical dystonia: results of a cross-sectional study. Park Relat Disord.

[23] Likert RA (1932). A technique for the measurement of attitudes. Arch Psychol.

[24] Bigalke H, Rummel A (2015). Botulinum neurotoxins: qualitative and quantitative analysis using the mouse phrenic nerve hemidiaphragm assay (MPN). Toxins.

[25] Dressler D, Bigalke H (2002). Botulinum toxin type A titres after cessation of botulinum toxin therapy. Mov Disord.

[26] Walter U, Mühlenhoff C, Benecke R (2020). Frequency and risk factors of antibody-induced secondary failure of botulinum neurotoxin therapy. Neurology.

